# Risk Factors for Relapse and Surgical Intervention in Children with CTEV Treated by Ponseti Method

**DOI:** 10.3390/life16030483

**Published:** 2026-03-16

**Authors:** Kamal Jamil, Yee Suan Goh, Siti Aqilah Aina Azman, Dharrsini Sathis, Baskaran Ramachandran, Gordon Nuil Grippin, Ahmad Fazly Abd Rasid

**Affiliations:** 1 Department of Orthopaedics & Traumatology, Faculty of Medicine, Universiti Kebangsaan Malaysia, Cheras, Kuala Lumpur 56000, Malaysia; a188757@siswa.ukm.edu.my (Y.S.G.); a179246@siswa.ukm.edu.my (S.A.A.A.); a187796@siswa.ukm.edu.my (D.S.); a188975@siswa.ukm.edu.my (B.R.); a189445@siswa.ukm.edu.my (G.N.G.); 2 Orthopaedic Unit, Hospital TABTAR (Children’s Specialist Hospital), Universiti Kebangsaan Malaysia, Cheras, Kuala Lumpur 56000, Malaysia; aleq017@gmail.com

**Keywords:** CTEV, Ponseti casting, relapse, surgical management, clubfoot

## Abstract

The Ponseti method is the gold standard for managing congenital talipes equinovarus (CTEV); however, relapses leading to surgical intervention remain a significant challenge. We investigated the factors associated with a higher risk of relapse. A retrospective study of 31 children (≤4 years) with CTEV treated with the Ponseti method between January 2014 and December 2023 was conducted. Demographic and clinical data—age at treatment, aetiology, Pirani score, number of casts, percutaneous Achilles tenotomy (PAT), surgery, bracing, relapses, and follow-up—were collected. Descriptive statistics, univariate analyses, and logistic regression were conducted to identify risk factors for relapse. Of the 31 patients, 67.7% were male, 58.1% had bilateral involvement and 71.0% had idiopathic CTEV. The median age at presentation was 2 months (IQR 1–3), and the initial Pirani score 5.0, improving to 0.0 at final follow-up (*p* < 0.001). The median number of casts was 7 (IQR 5–10), which correlated with the initial Pirani score (*p* = 0.021). PAT was performed in 77.4%; surgical intervention was required in 32.3%, most commonly repeat tenotomy (41.2%). The median bracing duration was 84 weeks (IQR 32–136) with 64.5% compliance. Relapses occurred in 35.5% of cases; 72.7% required recasting and surgery. Logistic regression identified brace compliance (*p* < 0.001) as the only significant factor; compliant patients had 90% lower odds of relapse. Non-compliance with bracing significantly increases the risk of relapses and surgical intervention in children with CTEV treated by the Ponseti method. Close monitoring and strict adherence to bracing are essential for an optimal outcome.

## 1. Introduction

Congenital talipes equinovarus (CTEV), or clubfoot, is one of the most common congenital deformities of the lower limb characterized by the inward turning and adduction of the foot and ankle. Clinically, this condition presents with four key deformities: forefoot adduction, mid-foot cavus, hindfoot varus, and equinus, each contributing to the overall structural abnormality. It can occur in isolation (idiopathic) or be associated with syndromic or neuromuscular conditions (non-idiopathic). The global incidence is estimated at 1.18 per 1000 live births, with male predominance and bilateral involvement in approximately half of cases [1,2]. Low-income countries recorded around 0.5 to 2.0 cases per 1000 live births [2]. In Malaysia, a study in 1990 reported a relatively high rate of 4.5 cases per 1000 live births [3], making it a notable healthcare concern. If left untreated, CTEV may cause permanent disability, leading to pain and difficulty when walking. Even with treatment, some patients may experience persistent functional limitations, such as restricted ankle mobility and muscle weakness, which can predispose them to early degenerative joint changes [4]. Therefore, CTEV may impact the quality of life, leading to significant psychosocial and economic consequences [2].

The Ponseti method, which involves gentle manipulation, serial casting, percutaneous Achilles tenotomy (PAT) and maintenance bracing, has become the gold standard for CTEV correction globally today [5,6]. The method is effective in producing a plantigrade, pain-free, and functional foot in the majority of cases, thus minimizing the need for extensive surgical procedures [5]. The correction process begins with casting to address cavus by supinating the forefoot to align with the hindfoot. Subsequent casts gradually correct adduction and varus by abducting the foot under the talus while applying counter pressure to prevent talus rotation. Once the foot reaches about 60–70° abduction with less than 15–20° dorsiflexion and other deformities corrected, a percutaneous Achilles tenotomy (PAT) is performed to treat equinus. This minor procedure is followed by casting in maximum dorsiflexion and abduction for about three weeks to maintain correction. After casting, a foot abduction brace (FAB) is used to maintain correction and prevent relapse. The brace consists of shoes joined by a bar that keeps the feet abducted and externally rotated. It is worn full-time (23 h/day) for the first three months, then gradually reduced to night use until the age of 3–4 years [7].

However, despite its proven efficacy [1,7,8,9,10,11], relapse and the need for further surgery post-method remain a recognized challenge. The relapse rates for CTEV treated with the Ponseti method range from 8.8% to 64.2% depending on various factors such as compliance with bracing and follow-up visits [11,12]. This wide variation in relapse rates may be attributed to differences in the definition of relapse and the duration of follow-up, with longer follow-up periods generally associated with higher rates over time [13]. Reported risk factors include poor brace compliance, socioeconomic deprivation, inadequate evertor muscle strength, and variable responses to initial casting [12,14,15,16]. However, despite this body of evidence, there remains a paucity of local data examining these determinants within our population, particularly in the context of diverse cultural and socioeconomic backgrounds. Therefore, apart from analyzing the outcome of the Ponseti method, this study aims to explore the risk factors associated with relapse and the need for surgical intervention among children with CTEV in our centre. We hypothesize that, in addition to brace compliance, factors such as demographic background, performing a tenotomy, casting duration, and initial treatment response significantly influence relapse risk, and that identifying these factors early can guide stratified treatment protocols, improve adherence strategies, and refine local management guidelines to optimize long-term functional outcomes.

## 2. Materials and Methods

### 2.1. Study Design

This retrospective observational study was conducted at a tertiary referral centre in Kuala Lumpur. The study aimed to determine the treatment outcomes and explore the risk factors for relapse and surgical intervention among children with congenital talipes equinovarus (CTEV) who were treated using the Ponseti method. Ethical approval was obtained from the Universiti Kebangsaan Malaysia Research Ethics Committee (JEP 2025-311). Informed consent from patients or guardians was not required, as only anonymized secondary data were analyzed.

### 2.2. Inclusion Criteria

Children aged four years and below who had been diagnosed with CTEV and treated primarily with the Ponseti casting method between January 2014 and December 2023 were included. A minimum follow-up duration of at least one year is required.

### 2.3. Exclusion Criteria

**Figure 1:**
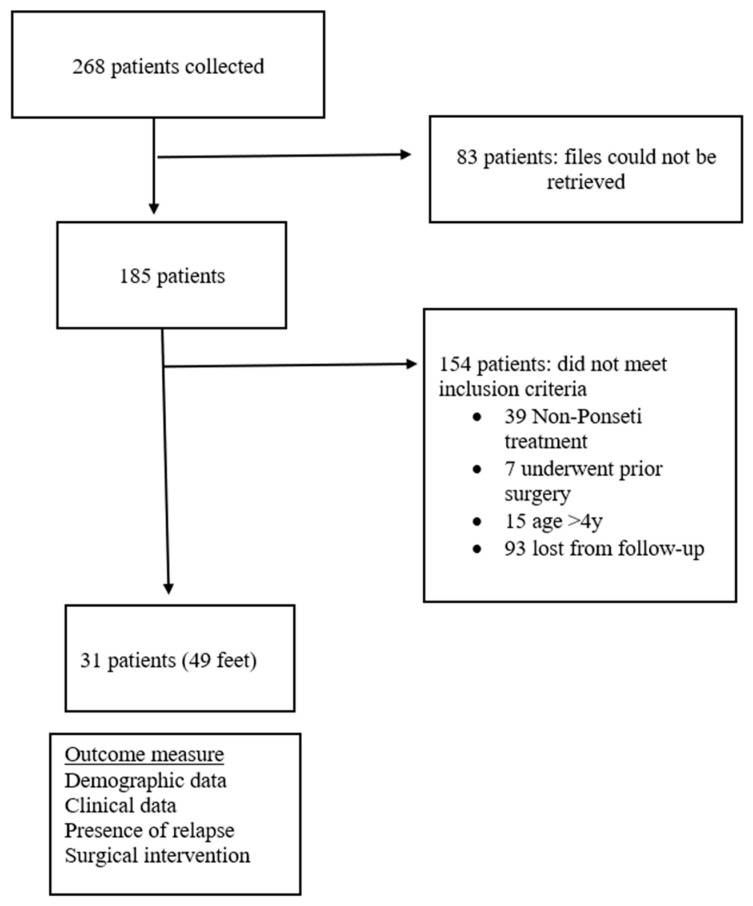
Flowchart of the patient selection and study design.

Patients with prior surgical correction, non-Ponseti management, or incomplete records were excluded through complete case analysis. Eligible cases were identified through the medical record databases. Figure 1 shows the flowchart of the patient selection and study design.

### 2.4. Treatment Method

All patients had been managed in accordance with the standard Ponseti protocol. Serial manipulation and plaster of Paris casting were performed weekly until satisfactory correction was achieved. If residual equinus deformity persisted after the correction of cavus, adductus, and varus, percutaneous Achilles tenotomy was performed under aseptic conditions. Following correction, a foot abduction brace was prescribed to be worn for 23 h per day during the first three months and subsequently during sleep until four years of age. Parents were counselled regarding brace care, and compliance was assessed during follow-up visits, mainly through parental reporting of hours of brace wear per day and consistency of brace usage [17].

### 2.5. Outcome Measures

Demographic data, including age at first presentation, sex, ethnicity, and laterality, were obtained. Clinical data collected included aetiology (idiopathic or non-idiopathic), Pirani score, total number of casts applied, need for percutaneous Achilles tenotomy, duration of bracing and compliance with brace usage.

The primary outcome measure was the Pirani score during treatment, which was recorded at baseline, one-year follow-up, and at the final visit. For bilateral cases, the mean Pirani score of both feet was used. Secondary outcome measures were the presence of relapse and need for surgical intervention, which were detected during the follow-up visits. Relapses were defined as recurrence of one or more components of the clubfoot deformity (cavus, adductus, varus, or equinus) that required further casting or surgical correction. Surgical intervention was defined as any operative procedure performed after the completion of Ponseti casting beyond the standard percutaneous Achilles tenotomy.

### 2.6. Statistical Analysis

Based on the data from a previous study on factors associated with relapse in Ponseti-treated congenital clubfoot conducted at Guilin Peoples Hospital [17], we gathered that the anticipated means of the significant values obtained were the number of casts between relapsed patients (7.15  ±  3.05) and non-relapse patients (5.20  ±  1.67). The sample size was then determined by utilizing a 2 means hypothesis testing formula, with the following parameters: 80% power with an expected difference of 0.2, a standard deviation of 0.55, a significance level of 0.05 and a 10% dropout rate. The normality assumption was tested using the Shapiro–Wilk test, considering the small sample size.

Data were analyzed using IBM SPSS Statistics for Windows, Version 30.0 (IBM Corp., Armonk, NY, USA). Continuous variables were presented as medians and interquartile ranges (IQRs), while categorical variables were expressed as frequencies and percentages. The Wilcoxon signed-rank test was used to compare pre- and post-treatment Pirani scores. Spearman’s correlation test was used to determine the association between continuous variables such as the initial Pirani score and the number of casts applied. Logistic regression analysis was performed to identify independent predictors of relapse and surgical intervention. As an exploratory measure, variables with a *p*-value < 0.25 in bivariate analysis were entered into the multivariate model. A *p*-value < 0.05 was considered statistically significant.

## 3. Results

A total of 31 children (49 feet) with CTEV treated using the Ponseti casting method were included in the study. Among the 31 patients, the majority were male (21; 67.7%), while 10 patients (32.3%) were female. Most patients were of Malay ethnicity (26; 83.9%), followed by Chinese (4; 12.9%) and Indian (1; 3.2%). A total of 18 patients (58.1%) presented with bilateral involvement, while 13 (41.9%) had unilateral involvement. In this cohort of 31 patients, the median age at first presentation was 2 months (IQR 1–3). The age ranged from 1 to 16 months, with the most common age being 1 month, accounting for 48.4% of cases. Overall, the distribution of ages was skewed towards the early months of life, with the majority presenting before 4 months of age.

Out of the 31 children, 22 (71.0%) or 34 feet had idiopathic CTEV, while 9 (29.0%) or 15 feet presented with non-idiopathic CTEV. Among the non-idiopathic cases, the vast majority were associated with arthrogryposis (66.7%) as an isolated condition. Spina bifida occulta (11.1%), Edward’s syndrome (11.1%) and Pierre Robin syndrome (11.1%) were much less frequent.

**Figure 2:**
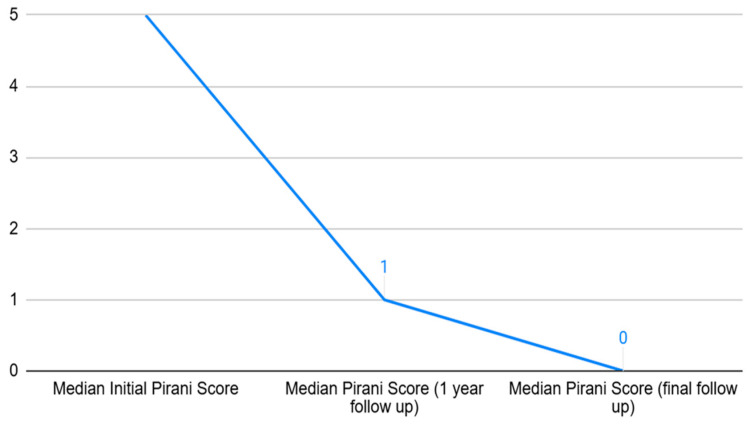
Pirani score values over the duration of follow-up (n = 31).

Since the sample size was less than 50, the Shapiro–Wilk test was used to assess normality, and all Pirani score variables were non-normally distributed (*p* < 0.05). Thus, data were presented as median (IQR), and comparisons were performed using the Wilcoxon signed-rank test. We found that Pirani scores decreased significantly following Ponseti casting. There was a significant reduction in Pirani scores from before casting (median 5.0, IQR 3.25–5.5) to 1-year follow-up (median 1.0, IQR 0.0–3.0) (Z = −4.799, *p* < 0.001). However, between the 1-year (median 1.0, IQR 0.0–3.0) and final follow-up (median 0.0, IQR 0.0–1.0), 15 patients improved further, 6 worsened, and 10 showed no change, although the change was significant (Z = −2.948, *p* = 0.003). When comparing the Pirani score over 3 time intervals, a decreasing trend was observed (Figure 2). The median duration of follow-up was 3 years (IQR 1.54–4.00).

**Figure 3:**
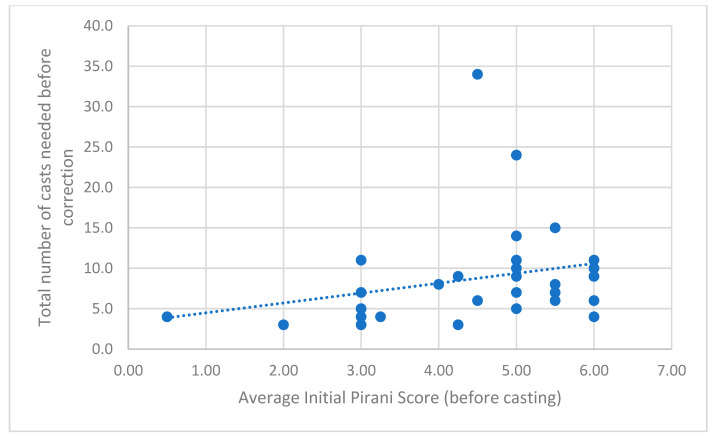
A higher initial Pirani score is associated with increased number of casts (n = 31).

The median number of casts required was 7.0 (IQR 5.0–10.0). The median age at the initiation of treatment was 1.00 month (IQR 1.00–3.00). There was no significant relationship between the age at which treatment was initiated and the total number of casts required before correction (Spearman’s ρ = −0.155, *p* = 0.404). Although children with non-idiopathic CTEV required slightly more casts (median = 9.0, IQR 4.0–10.25) compared to idiopathic cases (median = 7.0, IQR 4.00–10.50), this difference was not statistically significant (U = 89.00, Z = −0.437, *p* = 0.662). A significant moderate positive correlation was observed between the initial Pirani score and the total number of casts required (Spearman’s ρ = 0.412, *p* = 0.021) (Figure 3).

Out of 31 patients, 24 patients (77.4%) or 38 feet needed to undergo percutaneous Achilles tenotomy and 7 patients (22.6%) or 11 feet did not. The median age at the initiation of treatment was 2.00 months (IQR 1.0–3.0) for patients who underwent percutaneous Achilles tenotomy and 1.00 month (IQR 1.0–2.0) for those who did not. The difference in age between the two groups was not statistically significant (U = 54.5, Z = −1.511, *p* = 0.131). The proportion of patients requiring tenotomy was high in both idiopathic (77.3%) and non-idiopathic cases (77.8%). There was no significant association between aetiology and tenotomy requirement (Fisher’s Exact Test, *p* = 1.000).

The median duration of bracing was 62.00 weeks (IQR 28–130) for idiopathic CTEV patients and 104 weeks (IQR 40–192) for non-idiopathic patients. Although the non-idiopathic group appeared to have a longer bracing period, the difference was not statistically significant (U = 73.00, Z = −1.133, *p* = 0.257). There was no significant correlation between age at the initiation of treatment and duration of bracing (Spearman’s ρ = 0.047, *p* = 0.802). Spearman’s rank correlation showed no significant correlation between initial Pirani scores and duration of casting (Spearman’s ρ = −0.126, *p* = 0.499).

In total, 67.74% of the children (21 out of 31) or 32 feet were compliant with brace usage, whereas 32.26% of the children or 17 feet were reported to have low compliance. Patients who were non-compliant required more casts for correction (median = 8.5) compared to compliant patients (median = 6). However, the difference is not significant (U = 77, Z = −1.188, *p* = 0.235). Bracing compliance was observed in 63.6% of idiopathic cases and 77.8% of non-idiopathic cases. However, the difference was not statistically significant. There was no significant difference in age at initiation between compliance groups (median = 2.00 vs. 1.00 months; U = 95.5, Z = −0.649, *p* = 0.516). The initial Pirani score did not differ significantly between compliance groups (median 4.5 vs. 5.0; U = 75.00, Z = −1.464, *p* = 0.335).

**Figure 4:**
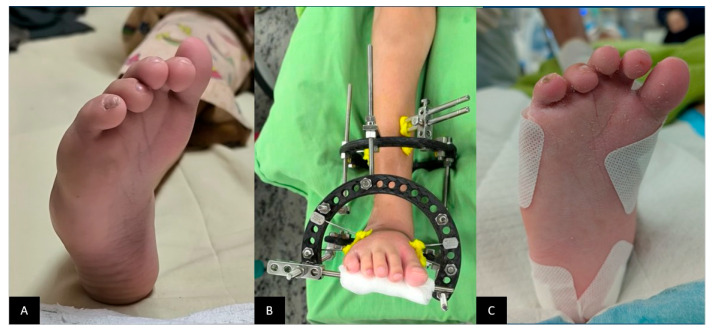
(**A**) Right foot relapsed CTEV at the age of 4 years. (**B**) An Ilizarov external fixator was applied after a series of casts. (**C**) The appearance of the foot following gradual correction.

At the final follow-up, 20 patients (64.5%) or 31 feet had no relapse following Ponseti casting. Relapse occurred in 5 patients (16.1%) affecting one foot and in 6 patients (19.4%) affecting both feet. Of the 11 patients who experienced relapse, 1 (9.1%) was managed with repeated casting alone, 2 (18.2%) underwent surgery alone, while the majority, 8 patients (72.7%), required a combination of repeated casting and surgical intervention. Among the types of surgeries performed, repeat percutaneous Achilles tenotomy was the most common at 41.2%, followed by posteromedial release (17.6%), Ilizarov external fixation (11.8%), and tibialis anterior tendon transfer (11.8%). Less common surgeries included talectomy and Achilles tendon lengthening, each done in 1 patient, accounting for 5.9% of the patients. Figure 4 shows an example of a patient who received treatment for relapse.

**Figure 5:**
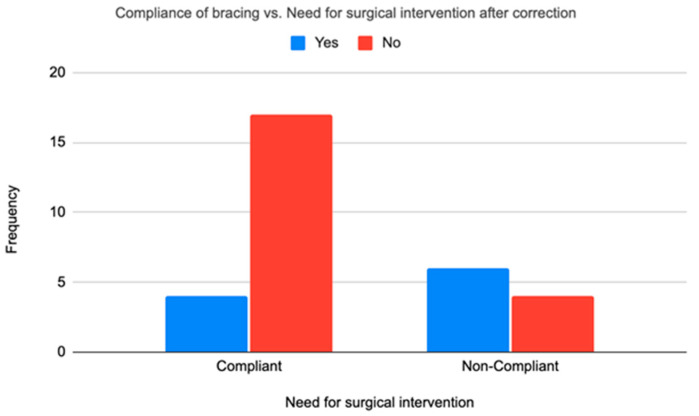
Relationship between the need for surgical intervention after casting was completed and compliance with bracing (n = 31).

We assessed the relationship between brace compliance and surgery. Seventeen out of 21 compliant patients (81.0%) or 22 feet did not require surgical intervention, while four patients (19.0%) or 8 feet still underwent surgery. In contrast, among non-compliant patients, 4 out of 10 patients (40%) or 8 feet avoided surgery and 60% or 9 feet required it. The association between bracing compliance and the need for surgical intervention was statistically significant (Fisher’s exact test, *p* = 0.040). (Figure 5).

All potential risk factors for relapse were first analyzed individually against relapse occurrence using appropriate univariate tests (Fisher’s exact test for categorical variables, Mann–Whitney U test for continuous variables) (Table 1). Variables with a *p*-value < 0.25 were then entered into binary logistic regression to further assess their association with relapse as an exploratory measure. These included ethnicity (*p* = 0.104), initial Pirani score (*p* = 0.161), tenotomy (*p* = 0.013) and compliance with bracing (*p* = 0.013). Odds ratios (ORs) with 95% confidence intervals (CIs) were then calculated, and statistical significance was set at *p* < 0.05 (Table 2).

## 4. Discussion

This study confirms the efficacy of the Ponseti method in our cohort, as reflected by a significant improvement in Pirani scores from baseline to final follow-up. Compared to surgery as the primary treatment, this method has consistently demonstrated superior outcomes in numerous previous studies [18,19,20,21]. However, relapse emerged as a major challenge, affecting up to 35% of our cases. This finding is similar to a prospective study by Halanski et al., who reported 15 out of 40 feet having relapse following Ponseti treatment [18]. It should be emphasized that not all instances of relapse necessitate major surgical intervention. Repeat casting would be the first step, followed by soft tissue surgeries for minor recurrences. A more severe or recalcitrant deformity would require joint-invasive procedures, e.g., posteromedial release as treatment [13]. Other studies reported a lower incidence of relapse [1,8,9], but some had shorter follow-up [11] and a younger population [22] of patients. Although non-idiopathic CTEV has been reported to respond less favourably to the Ponseti method [23], our findings did not indicate an increased relapse rate within this subgroup.

Our findings add valuable local context to treatment outcomes and relapse patterns. Relapse was noted even beyond the first year of treatment, emphasizing the importance of prolonged follow-up and surveillance. The number of casts required for initial correction in our cohort was slightly higher than those reported by other authors [10,23], likely reflecting more severe baseline deformities typical of tertiary referrals, as well as some syndromic cases. This interpretation is supported by the positive correlation between initial Pirani scores and total casts required, reinforcing the established understanding that greater deformity severity necessitates more manipulations to achieve correction [23].

The frequency of PAT in our cohort (77.4%) aligns with the increasingly widespread adoption of the procedure, as evidenced in multiple studies with rates ranging from 23.1% to 90.4%, depending on the type of clubfoot and population studied [9,22]. Despite the overall success of initial correction, 35.5% of patients ultimately required surgical intervention, with repeat PAT being the most common procedure. This reflects equinus as the most frequent and difficult component of relapse to maintain. The limited success of repeat casting for recurrent deformities in our cohort further supports the notion that relapsed CTEV often becomes more rigid and less amenable to conservative treatment, making early prevention through compliance crucial.

We found that brace non-compliance is associated with further surgery. Our exploratory analysis revealed that brace non-compliance was the only statistically significant predictor of relapses. Compliant patients demonstrated substantially lower odds of recurrence (90% reduction) consistent with findings from Hu et al. [17], though our results suggest that brace adherence outweighs other risk factors such as age at first casting or initial severity. Similarly, in a study by Halanski et al. [18], the severity of the initial deformity and the age at which treatment began did not have a significant effect on the risk of relapse. These results reaffirm that caregiver adherence, shaped by psychosocial, educational, and practical factors, is the pivotal determinant of long-term success. Evidence-based bracing regimens typically recommend full-time use for 2–3 months [21] or up to 6 months [19], followed by nighttime bracing for 2–5 years [8,24]. Apart from that, persistent issues related to brace fitting, sizing, and follow-up coordination remain practical barriers that may contribute to relapse [16]. Strengthening caregiver education, structured brace-monitoring protocols, and rapid adjustment services may therefore play a crucial role in improving outcomes.

The demographic and clinical profile of our cohort closely mirrors previous studies, with male predominance, bilateral involvement, and similar racial distribution patterns [20,25,26]. This study contributes to the limited Malaysian literature on relapse risk factors in CTEV by analysing a real-world cohort from a tertiary centre using standardized Pirani scoring. The clear identification of brace non-compliance as the central determinant of relapse provides actionable guidance for clinicians, emphasising the need for intensive parental counselling, structured long-term follow-up, and improved brace-related support systems.

Nevertheless, our study has inherent limitations. The retrospective design, limited sample size, referral bias inherent to a tertiary care setting, and heterogeneous follow-up durations restrict generalisability and may disproportionately represent more severe cases. Brace compliance was evaluated based on subjective parental reports, which may introduce recall bias, while the involvement of multiple assessors during follow-up could compromise the consistency and reliability of the findings. We were unable to locate a large number of patients who were lost to follow-up, which may lead to attrition bias. Future prospective, multicentre studies with larger samples and long-term functional outcome measures such as gait and pain assessment are warranted to validate and extend these findings.

## 5. Conclusions

Children who adhered to the prescribed foot abduction bracing protocol potentially have lower odds of recurrence and a subsequently reduced need for repeat interventions. This underscores the crucial role of long-term brace adherence in maintaining correction and avoiding relapses, particularly during early childhood when deformity progression is most likely. Innovations in brace technology—such as flexible designs, advanced materials, and embedded sensors—may represent a future direction for enhancing treatment compliance.

## Figures and Tables

**Table 1:** Univariate analysis of all potential risk factors for relapse. (n = 31).

Variables	Relapse Clubfeet	Normal Clubfeet	Statistical Test	*p*-Value
Gender			Fisher’s exact	0.262
Male	9	12		
Female	2	8		
Ethnicity			Fisher’s exact	0.104 *
Malay	10	16		
Chinese	0	4		
Indian	1	0		
Aetiology			Fisher’s exact	1.000
Idiopathic	8	14		
Non-idiopathic	3	6		
Affected leg			Fisher’s exact	0.275
Bilateral	8	10		
Unilateral Right or Left	3	10		
Age at 1st presentation (month)	2.00	1.00	Mann–Whitney U	0.489
	(1.00–4.00)	(1.00–2.75)		
Age at initiation of treatment (month)	2.00	1.00	Mann–Whitney U	0.576
	(1.00–3.00)	(1.00–2.00)		
Initial Pirani score	5.00	4.50	Mann–Whitney U	0.161 *
	(4.50–6.00)	(3.00–5.50)		
Number of casts for correction (1st cycle)	6.00	6.00	Mann–Whitney U	0.467
	(5.00–9.00)	(4.00–8.75)		
Tenotomy			Fisher’s exact	0.013 *
Yes	10	14		
No	0	7		
Walking age (months)	20.50	18.00	Mann–Whitney U	0.529
	(16.75–36.75)	(18.00–24.00)		
Duration of bracing (weeks)	96.00	62	Mann–Whitney U	0.664
	(28.00–180.00)	(45.00–123.00)		
Compliance to brace usage			Fisher’s exact	0.013 *
Yes	4	17		
No	7	3		

* *p* < 0.25.

**Table 2:** Binary logistic regression of all potential risk factors for relapses (n = 31).

Variables	Sig.	Odds Ratio	95% Confidence Interval for Odds Ratio
Ethnicity	1.000	<0.001	<0.01–999.9
Initial Pirani score	0.127	0.571	0.278–1.173
Need for tenotomy	0.999	1,366,940,252.00	<0.01–999.9
Compliance with brace usage	0.010 *	0.101	0.018–0.573

* *p* < 0.05.

## Data Availability

Restrictions apply to the availability of these data. Data were obtained from Pusat Informatik Kesihatan (PIK) Hospital Canselor Tuanku Muhriz (HCTM) and are available from the authors with the permission of Pusat Informatik Kesihatan (PIK) Hospital Canselor Tuanku Muhriz (HCTM).

## References

[1] Bitew A., Melesse D.Y., Admass B.A. (2023). A 5-years results of the Ponseti method in the treatment of congenital clubfoot: A retrospective study. Eur. J. Orthop. Surg. Traumatol..

[2] Smythe T., Rotenberg S., Lavy C. (2023). The global birth prevalence of clubfoot: A systematic review and meta-analysis. EClinicalMedicine.

[3] Boo N., Ong L. (1990). Congenital talipes in Malaysian neonates: Incidence, pattern and associated factors. Singap. Med. J..

[4] Saran J.S.R.G., Devdass V., Anand D. (2025). Understanding Clubfoot: Integrating Historical Origins, Embryologic Foundations, Epidemiology and Etiology—A Review. Birth Defects Res..

[5] Cooke S.J., Balain B., Kerin C.C., Kiely N.T. (2008). Clubfoot. Curr. Orthop..

[6] Ganesan B., Luximon A., Al-Jumaily A., Balasankar S.K., Naik G.R. (2017). Ponseti method in the management of clubfoot under 2 years of age: A systematic review. PLoS ONE.

[7] Colburn M.W.M. (2003). Evaluation of the treatment of idiopathic clubfoot by using the Ponseti method. J. Foot Ankle Surg..

[8] Asuquo J.E., Okokon E.O., Lasebikan O.A., Anisi C.O., Asuquo B.J., Abang I.E., Obaji A.E., Chigbundu K.C. (2024). Assessment of Treatment Outcomes in the Management of Club foot using the Ponseti Technique: A cross-sectional study. Afr. J. Paediatr. Surg..

[9] Bakarman K.A., Zamzam M.M., Addweesh A.K., Basalem S.M., Alsanad F.A., AlHamdi K.M., Alenezi T.M. (2024). Outcomes of clubfoot conservative treatment using the Ponseti technique in an academic hospital in Saudi Arabia. J. Musculoskelet. Surg. Res..

[10] Daun E., Bajuri M.Y., Abd Rashid A.H., Ibrahim S., Das S. (2018). Outcome of Ponseti Method in Treating Congenital Idiopathic Clubfoot: Five Years’experience at a Tertiary Hospital. Asian J. Pharm. Clin. Res..

[11] Gunalan R., Mazelan A., Lee Y., Saw A. (2016). Pattern of presentation and outcome of short-term treatment for idiopathic clubfoot/CTEV with Ponseti method. Malays. Orthop. J..

[12] Martinez A.S., Loyd G., Bridges C., Milad M., Pathare N., Doston L., Gugala Z., Hill J.F. (2024). Missed Visits Predict Recurrence in Idiopathic Clubfoot. J. Pediatr. Orthop..

[13] Thomas H.M., Sangiorgio S.N., Ebramzadeh E., Zionts L.E. (2019). Relapse rates in patients with clubfoot treated using the Ponseti method increase with time: A systematic review. JBJS Rev..

[14] Dobbs M.B., Rudzki J.R., Purcell D.B., Walton T., Porter K.R., Gurnett C.A. (2004). Factors Predictive of Outcome after Use of the Ponseti Method for the Treatment of Idiopathic Clubfeet. J. Bone Jt. Surg..

[15] Gelfer Y., Dunkley M., Jackson D., Armstrong J., Rafter C., Parnell E., Eastwood D.M. (2014). Evertor muscle activity as a predictor of the mid-term outcome following treatment of the idiopathic and non-idiopathic clubfoot. Bone Jt. J..

[16] Kuzma A.L., Talwalkar V.R., Muchow R.D., Iwinski H.J., Milbrandt T.A., Jacobs C.A., Walker J.L. (2020). Brace Yourselves: Outcomes of Ponseti Casting and Foot Abduction Orthosis Bracing in Idiopathic Congenital Talipes Equinovarus. J. Pediatr. Orthop..

[17] Hu W., Ke B., Niansu X., Li S., Li C., Lai X., Huang X. (2022). Factors associated with the relapse in Ponseti treated congenital clubfoot. BMC Musculoskelet. Disord..

[18] Halanski M.A., Davison J.E., Huang J.-C., Walker C.G., Walsh S.J., Crawford H.A. (2010). Ponseti method compared with surgical treatment of clubfoot: A prospective comparison. JBJS.

[19] Hallaj-Moghaddam M., Moradi A., Ebrahimzadeh M.H., Habibzadeh Shojaie S.R. (2015). Ponseti casting for severe club foot deformity: Are clinical outcomes promising?. Adv. Orthop..

[20] Lee B., Mazelan A., Gunalan R., Albaker M., Saw A. (2020). Ponseti method of treating clubfoot-Is there difference if treatment is started before or after one month of age?. Med. J. Malaysia.

[21] Saini R., Sharma A., Ravalji D., Baisoya K., Sharma G. (2023). A prospective study on functional outcomes of serial cast correction in congenital Talipes equinovarus (CTEV) by Ponseti method. Cureus.

[22] Enweluzo G.O., Ohadugha A.G., Ezenwa-Ahanene I.C., Udechukwu O.I., Edem U.I. (2024). Management outcome of congenital talipes equinovarus (clubfoot) using Ponseti protocol at Lagos University Teaching Hospital. J. West Afr. Coll. Surg..

[23] Sharma P.K., Verma V., Meena S., Singh R., Km P. (2021). Comparative evaluation and analysis of outcomes in non-idiopathic and idiopathic clubfeet with Ponseti method at a tertiary care centre of a developing country. Foot.

[24] Dreise M., Elkins C., Muhumuza M.F., Musoke H., Smythe T. (2023). Exploring bracing adherence in ponseti treatment of clubfoot: A comparative study of factors and outcomes in Uganda. Int. J. Environ. Res. Public Health.

[25] Alyana N., Sahdi H., Rasit A., Zabidah P. (2018). Barriers affecting Clubfoot treatment in Sarawak. J. Health Transl. Med. (JUMMEC).

[26] Rasit A., Azani H., Zabidah P., Merikan A., Alyana B.N. (2012). Clubfoot: The treatment outcome using quantitative assessment of deformity. Malays. Orthop. J..

